# Maternal effects of the English grain aphids feeding on the wheat varieties with different resistance traits

**DOI:** 10.1038/s41598-018-25136-x

**Published:** 2018-05-09

**Authors:** Xiang-Shun Hu, Zhan-Feng Zhang, Tong-Yi Zhu, Yue Song, Li-Juan Wu, Xiao-Feng Liu, Hui-Yan Zhao, Tong-Xian Liu

**Affiliations:** State Key Laboratory for Crop Stress Biology in Arid Areas, Key Laboratory of Crop Pest Management on the Northwest Loess Plateau of Ministry of Agriculture, College of Plant Protection, Northwest A & F University, Yangling, Shaanxi 712100 China

## Abstract

The maternal effects of the English grain aphid, *Sitobion avenae* on offspring phenotypes and performance on wheat varieties with different resistance traits were examined. We found that both conditioning wheat varieties(the host plant for over 3 months) and transition wheat varieties affected the biological parameters of aphid offspring after they were transferred between wheat varieties with different resistance traits. The conditioning varieties affected weight gain, development time (DT), and the intrinsic rate of natural increase (r_m_), whereas transition varieties affected the fecundity, r_m_, net reproductive rate, and fitness index. The conditioning and transition wheat varieties had significant interaction effects on the aphid offspring’s DT, mean relative growth rate, and fecundity. Our results showed that there was obvious maternal effects on offspring when *S. avenae* transferred bwteen wheat varieties with different resistance level, and the resistance traits of wheat varieties could induce an interaction between the conditioning and transition wheat varieties to influence the growth, development, reproduction, and even population dynamics of *S. avenae*. The conditioning varieties affected life-history traits related to individual growth and development to a greater extent, whereas transition varieties affected fecundity and population parameters more.

## Introduction

Phenotypic plasticity is the ability of an organism to express multiple phenotypes with differences in physiology, morphology, or behavior in response to changes in environmental conditions^1–5^. Maternal effects represent the influence of environmental variation in the maternal generation on phenotypic differences in the offspring generation^6–8^. The mechanisms of maternal effects on offspring are always fundamentally related to the induction of enzymatic activity and molecular pathways, mRNA and protein expression, or hormone secretion. The products of molecular and biochemical activity are deposited into the zygotes^9^, which further influence biological characteristics, such as the incidence and intensity of diapause, production of sexual forms, wing polyphenism, dispersal behavior, development time, growth rate, resistance to chemicals or microbial infection, and survival in insects^6,10^. Aphid reproduction is typically parthenogenic for at least a part of their lifecycle^11^. Asexual mother aphids are genetically identical to their daughters and sisters, and form telescoping generations (embryos within embryos), so that granddaughters are present within the bodies of their grandmothers^12^. Therefore, strong maternal and transgenerational effects exist in aphids, which can extend across as many as three generations^6,11^. This lifecycle makes the aphid an ideal organism to study maternal effects on phenotypic plasticity.

Host plant is an important factor that influences aphid ecology, and it is therefore a primary target of research into maternal effects and phenotypic plasticity^13^. Maternal effects on aphid species have been documented on a wide variety of host plant species. For example, the performance of some *Rhopalosiphum maidis* populations was better on a novel host, wheat, than on the original host, Johnson grass (*Sorghum halepense* L.)^14^. Considering host plant preference, there is no evidence that the maternal host species influences the fecundity of offspring in the milkweed-oleander aphid (*Aphis nerii* Boyer de Fonscolombe) or the pea aphid (*Acyrthosiphon pisum* (Harris))^15,16^. For the bird cherry-oat aphid *R. padi*, the grandoffspring generation had higher mean relative growth rates (MRGRs) than those of the grandmother generation, as well as offspring of emigrants, and alate individuals from (*Prunus padus* L.) on both seedlings and flowers of oat^17^. However, the maternal effects in these studies focused on host alternation, an obligatory seasonal shifting between genetically distantly related host plants.

The English grain aphid *Sitobion avenae* (Fab.) (Hemiptera: Aphididae) is an important pest of wheat (*Triticum aestivum* L.) and other cereals throughout the world, causing severe economic damage^18–20^. The life-history traits of *S. avenae*, including the mode of reproduction and growth rate, display substantial heritable variation in relation to host plants^21,22^. The alatae host preference of *S. avenae* is marginally influenced by the aphid’s genotype, but is strongly influenced by the host species they feed on^23–25^. Resistant wheat varieties can increase aphid mortality, reduce aphid body size (weight), extend the development time (DT) of nymphs, (thereby increasing the risk of parasitism and/or predation), and decrease offspring production, all of which influence the rate of population increase^18,26,27^. When *R. padi* was maintained on the same wheat varieties for over 3 months, its life-history traits related to individual growth and development significantly declined, whereas those of *S. avenae* significantly improved. However, the fecundity of *S. avenae* were significantly decreased on the wheat varieties Batis, WW2730, and Xiaoyan22; the r_m_ on Batis and WW2730; and the net reproductive rates (NRRs) on Batis and Xiaoyan22 (our unpublished data). The transgenerational maternal effect on *R. padi* were showed that this species produced more alatae when maintained on resistant wheat varieties for 3 months, even when their offspring were maintained on a susceptible wheat variety. Furthermore, these alatae had a higher fitness index (FI) and r_m,_ and better performance on novel hosts than that of maternal hosts^10^.

In the present study, we investigated the transgenerational maternal effect of wheat varieties with different resistance traits on phenotypic plasticity of *S. avenae* offspring. This research could provide the basis for using the resistance of the host wheat variety to manage infestations of these aphids.

## Materials and Methods

### Aphids and Wheat Varieties

**Figure 1:**
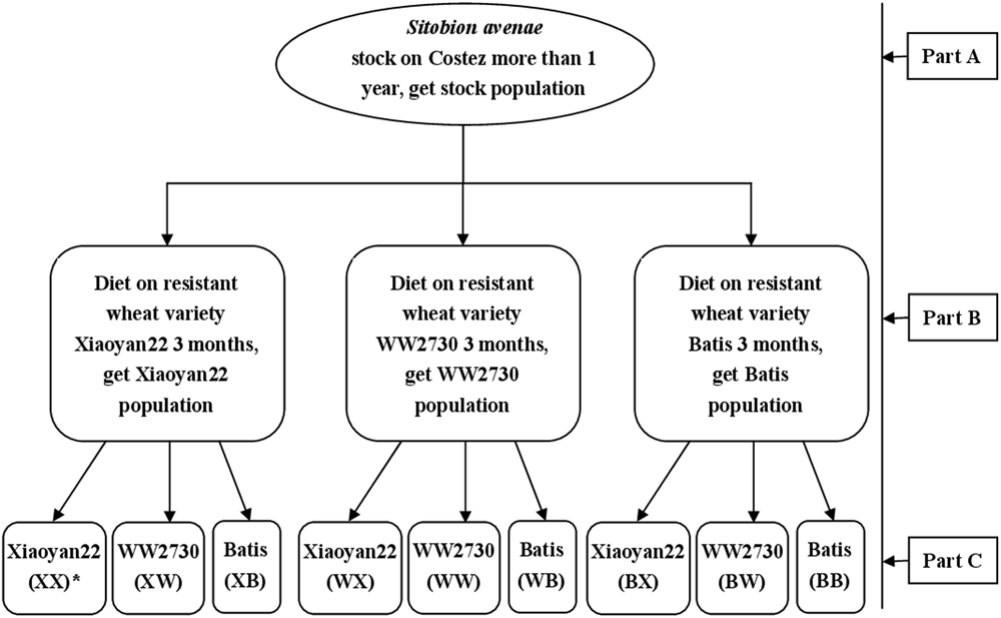
This figure show how the English grain aphid *Sitobion avenae*, were derived and how the three host-adapted strains were generated. Note: “*” The first capital letters in the brackets represented the acclimatized population (or conditioning wheat variety), and the second represented the transferred transition wheat variety.

A stock population of *S. avenae*, which originated from an individual mother aphid collected from a wheat field near Braunschweig, Germany, was maintained on wheat seedlings (*cv*. Costez, a susceptible wheat variety) at a daytime temperature of 20 ± 0.5 °C, night temperature of 18 ± 0.5 °C, 16:8 h light: dark cycle, and approximately 70% relative humidity in a plant growth chamber for more than 1 year (Fig. 1A).

The host wheat varieties used were Xiaoyan22, with a chromosome from *Agropyron elongatum* (Host) Beauv (=*Elytrigia elongata*), originating from China, and WW2730 and Batis, originating from Germany. Xiaoyan22 and WW2730 are relatively resistant to aphids. Batis is susceptible to both *S. avenae* and *R. padi* at the seedling phase. Batis was used as a conditional variety^28^. The possible restriction factors of WW2730 against *S. avenae* are in the epidermis, mesophyll, and phloem, and those of Xiaoyan 22 are in the mesophyll^29^.

The wheat seedlings were planted in 9 × 9 × 10 cm plastic pots. The potting medium was a mixture of soil with sand, humus, and blank loamy soil at a ratio of 1:3:3. Wheat seedlings at the two-leaf stage(13 days after sowing) were used in all subsequent experiments.

### Experimental design

Approximately 30 apterous aphid adults were collected from the stock population and allowed to reproduce for 24 h on wheat seedlings (*cv*. Costez). Approximately 20 newborn first-instar nymphs (**<**24 h old) were individually transferred to seedlings of each of the three wheat varieties, Xiaoyan22, WW2730, or Batis, which were maintained in separate cages (50 × 50 × 50 cm). The cages were placed in a plant growth chamber with the same conditions as those of the stock population. These aphids were allowed to acclimatize for 3 months (equivalent to approximately 10 generations) to establish three acclimatized populations: Batis population, WW2730 population, and Xiaoyan22 population (Fig. 1B).

Approximately 50 apterous adults (mother aphids) from each acclimatized population were placed on new seedlings of the same variety as their parent population. The first-instar nymphs produced within 24 h (offspring aphids) by these apterous aphids were used in the experiments. Each experimental treatment involved transferring offspring aphids from one of the acclimatized populations to each of the three host wheat varieties (transition varieties). These treatments were marked with two capital letters: XX, XW, XB; WX, WW, WB; BX, BW, and BB. The first letter represents the acclimatized population (conditioning wheat variety), and the second represents the transition wheat variety (transition wheat varieties) (Fig. 1C).

Each treatment was replicated 30 times. A single first-instar nymph that was transferred to a single test wheat seedling in a pot was regarded as one replicate. The test wheat seedling with a single nymph was covered with a ventilated, clear-glass cylinder (4 cm in diameter and 24 cm in height) to prevent the aphids from escaping^30^. The weight of each single first-instar nymph (Wn), molting, weight of the newly molted adult (Wa), and the offspring number produced were monitored and recorded. A single aphid weight was weighed with an electronic balance (Sartorius MSA, Göttingen, Germany). The biological parameters of each aphid offspring were used to evaluate maternal effects (Table 1).

**Table 1:** The biological parameters were measured in this study.

Biological parameters (Abbr.)	Measurement
Nymphal survival (NS)	Adult number/first instar larvae number
Development time (DT)	The duration from birth to adult emergence +0.5 d (Thieme and Heimbach, 1996)
Weight gain (WG)	Wa – Wn^*^ (Thieme and Heimbach, 1996)
Fecundity	Offspring of per female produced within a duration that was equal to DT after reaching maturity (Thieme and Heimbach, 1996)
Mean relative growth rates(MRGR)	(lnWa - lnWn)/DT (Thieme and Heimbach, 1996)
The intrinsic rate of natural increase (r_m_)	0.738 ln(fecundity)/DT (Wyatt and White 1977; Leather and Dixon, 1984)
Net reproduction rate (NRR)	NS × fecundity/(2 × DT)
Fitness index (FI)	NRR × MRGR

^*^Wa is the newly adult weight emerged within 24 h, Wn is the first-instar nymph weight (newborn within 24 h).

### Data analysis

We used hierarchical-level analysis to analyze the data. First, the individual-level hierarchical traits, DT, weight gain (WG), and fecundity were compared across the conditioning and transition wheat varieties, as well as the interactive effects, by fixed effects two-way analysis of variance (ANOVA) using the generalized linear model (GLM). Second, the population-level hierarchical traits, nymph survival (NS), r_m_, NRR, MRGR, and FI were compared across the conditioning and transition wheat varieties using fixed effects two-way ANOVA based on the GLM. NS percentage data were transformed using the inverse sine transformation because most of the values were greater than 70%, and they were compared among conditioning and transition wheat varieties using a fixed effect two-way ANOVA without repeated measures^31^.

The homogeneity of variance of all parameters was tested before each analysis. Non-normally distributed data were log-transformed. When ANOVA results indicated a significant effect of a factor or an interaction, the means were separated using Tukey’s test at *P* < 0.05.

Three group specific comparisons of means originating from the interaction were compared using independent sample t-tests. In the first group were three comparisons, WX vs. BX, XW vs. BW and XB vs. WB, which have the same transition wheat varieties, but different conditioning wheat variety. The second group were three comparisons, XB vs. XW, WX vs. WB and BX vs. BW, different transition wheat varieties, but same conditioning wheat variety. In the third group were three comparisons, XW vs. WX, XB vs. BX and WB vs. BW, different conditioning varieties and a different transition variety reciprocally transferred.

All data were analyzed using SPSS version 17.0 software (IBM SPSS Inc., Chicago, IL, USA).

## Results

### Nymph survival, weight gain, development time and fecundity

*S. avenae* NS did not significantly differ between the conditioning and transition varieties (Table 2). The WG and DT were significantly different among the three conditioning varieties, whereas the fecundity differed significantly among the three transition varieties. The DT and fecundity were also significantly affected by interactions between the conditioning and transition varieties (Table 2).

**Table 2:** The analysis of variance results for *Sitobion avenae* life-history traits (3 conditioning wheat varieties ×3 transition wheat varieties).

Factors		Individual-level hierarchical traits				Population-level hierarchical traits			
		Development time	Weight Gain	Mean Relative Growth Rate	Fecundity	r_m_	Nymphal Survival	Net Reproduction Rate	Fitness Index
Conditioning varieties	*F* ratios	3.04	3.29	1.38	0.85	3.57	3.72	0.45	0.69
	*P* value	**0.05**	**0.04**	0.26	0.43	**0.03**	0.12	0.64	0.50
Transition varieties	*F* ratios	0.32	1.34	1.39	11.21	4.07	3.64	16.59	12.06
	*P* value	0.73	0.26	0.25	**<0.01**	**0.02**	0.13	**<0.01**	**<0.01**
Interaction	*F* ratios	7.78	0.84	5.16	2.72	1.45	#	1.21	1.20
	*P* value	**<0.01**	0.50	**<0.01**	**0.03**	0.22	#	0.31	0.31

Note: The degrees of freedom are all 2, 2, 4, 245.

**Figure 2:**
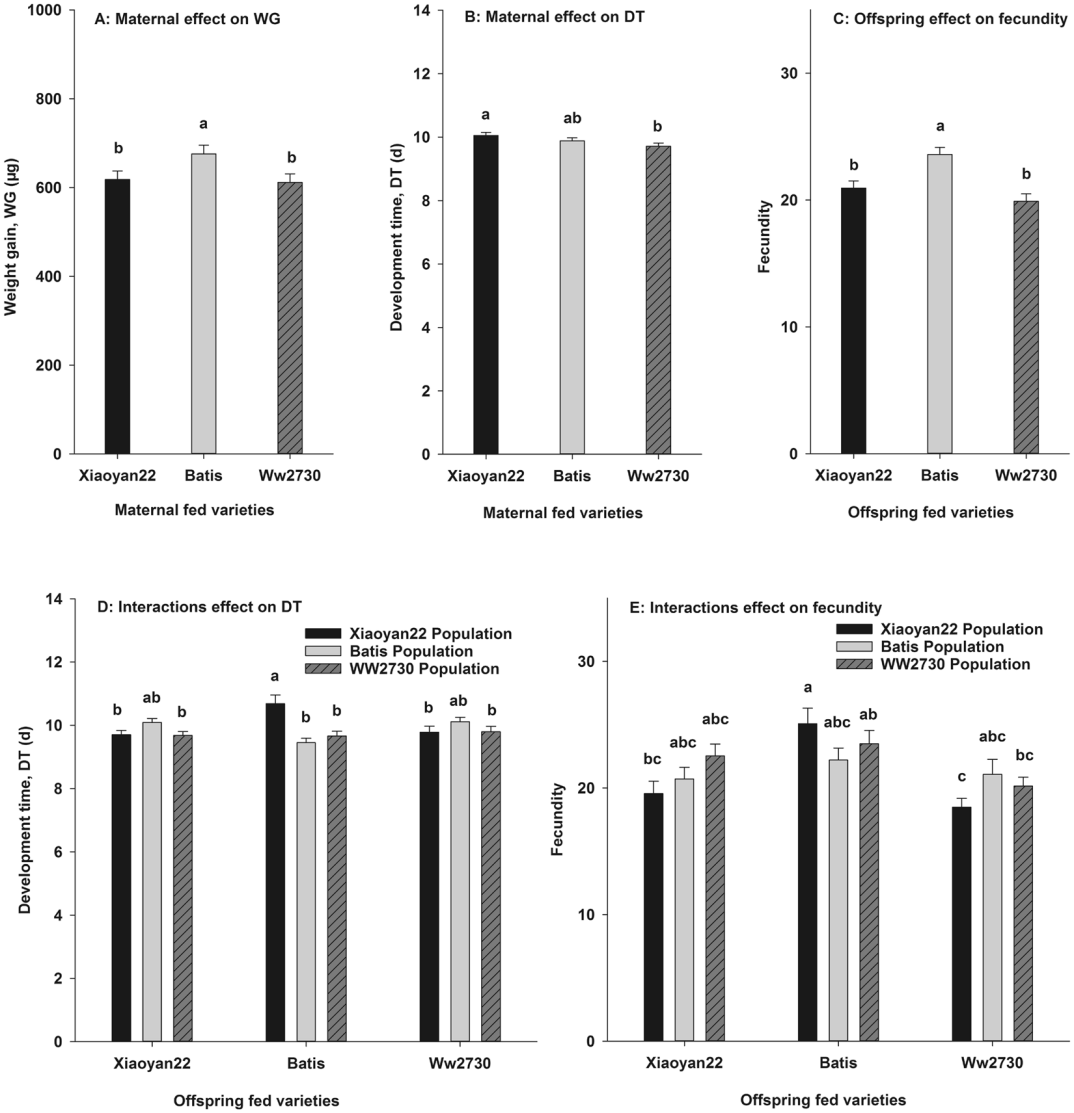
The individual-level hierarchical traits, nymphal survival (NS), development time (DT), weight gain (WG), and fecundity change of *Sitobion avenae* with host shifted.

The WG of *S. avenae* from the Batis population was significantly greater than that from the Xiaoyan22 or WW2730 population (Fig. 2A). The DT of *S. avenae* from the Xiaoyan22 population was significantly longer than that from the WW2730 population (Fig. 2B). The mean fecundity of offspring for the three acclimatized *S. avenae* populations transferred to the Batis was significantly greater than that of the population transferred to WW2730 or Xiaoyan22 (Fig. 2C).

The mean DT of individuals from the XB treatment (offspring from the conditioning Xiaoyan22 population transferred to the Batis host variety) was the longest, significantly longer than that from the XX, WX, BB, WB, XW, or WW treatments (Fig. 2D). The highest fecundity was observed in individuals in the XB treatment, and it was significantly higher than that in the XX, XW, or WW treatments. The fecundity was the lowest in the individuals of the XW treatment, which was significantly lower than that from the XB or WB treatment (Fig. 2E).

### Mean relative growth rates, intrinsic rates of natural increase, net reproductive rate, and fitness index

**Figure 3:**
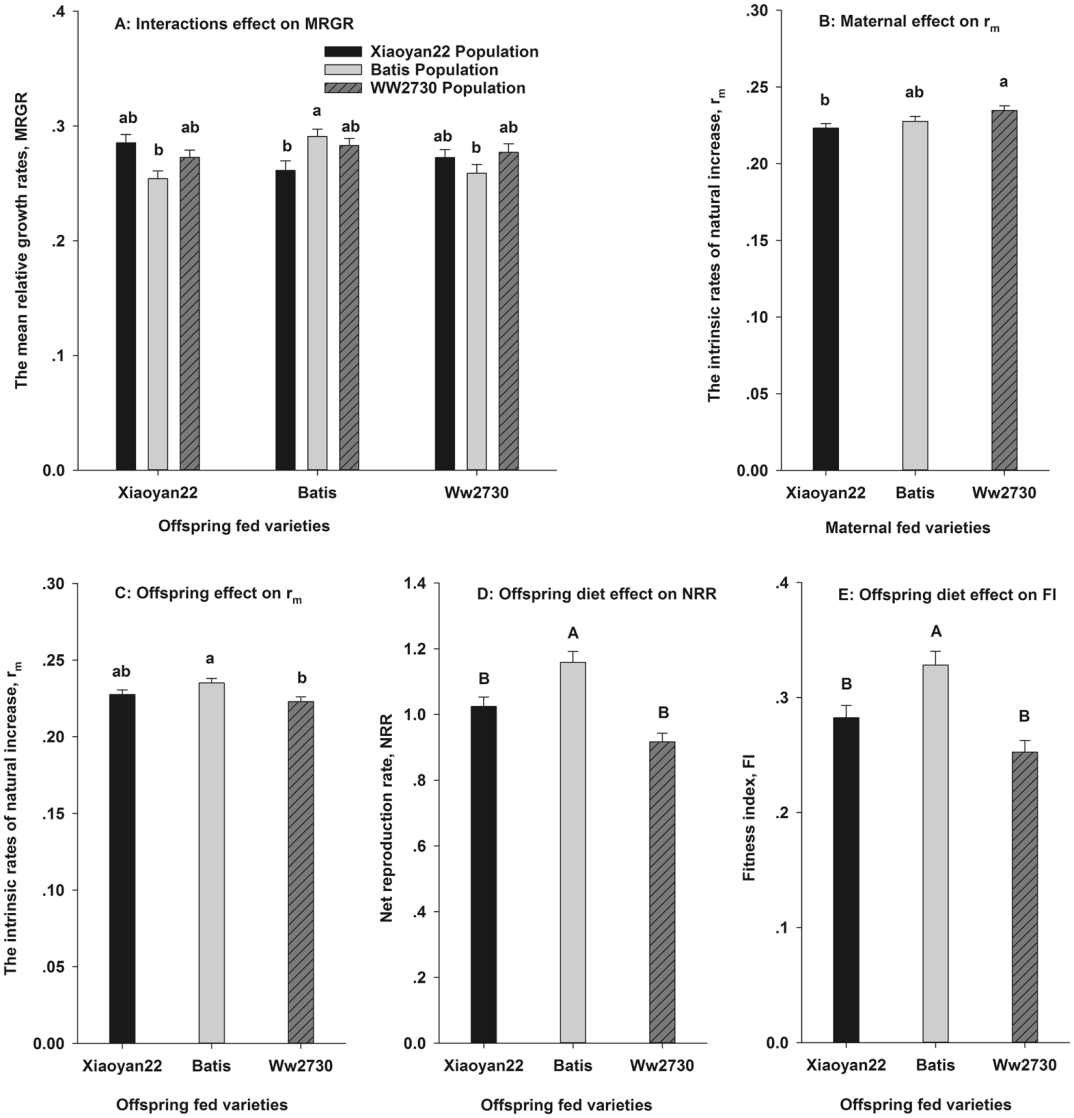
The population-level hierarchical traits, mean relative growth rates(MRGR), the intrinsic rate of natural increase (r_m_), net reproduction rate (NRR), and fitness index (FI) change of *Sitobion avenae* with host shifted.

The MRGRs of *S. avenae* were significantly affected by interactions between the conditioning varieties and transition varieties (Table 2). The highest MRGR of *S. avenae* was observed in the BB treatment (offspring from the conditioning Batis population transferred to Batis), and it was significantly greater than those of the XB, BX, or BW treatments (Fig. 3A).

The r_m_ values differed significantly among the three conditioning varieties and transition varieties, but there were no interactive effects (Table 2). The r_m_ values of *S. avenae* from the WW2730 population were significantly greater than those from the Xiaoyan22 population (Fig. 3B). The r_m_ of offspring transferred to WW2730 was significantly lower than that of offspring transferred to Batis (Fig. 3C).

The NRR and FI were significantly different among the three transition varieties (Table 2). Both the NRR and FI of *S. avenae* offspring transferred to Batis were greater than those of offspring transferred to Xiaoyan22 or WW2730 (Fig. 3D,E).

### Specific comparisons of means originating from the interaction

**Figure 4:**
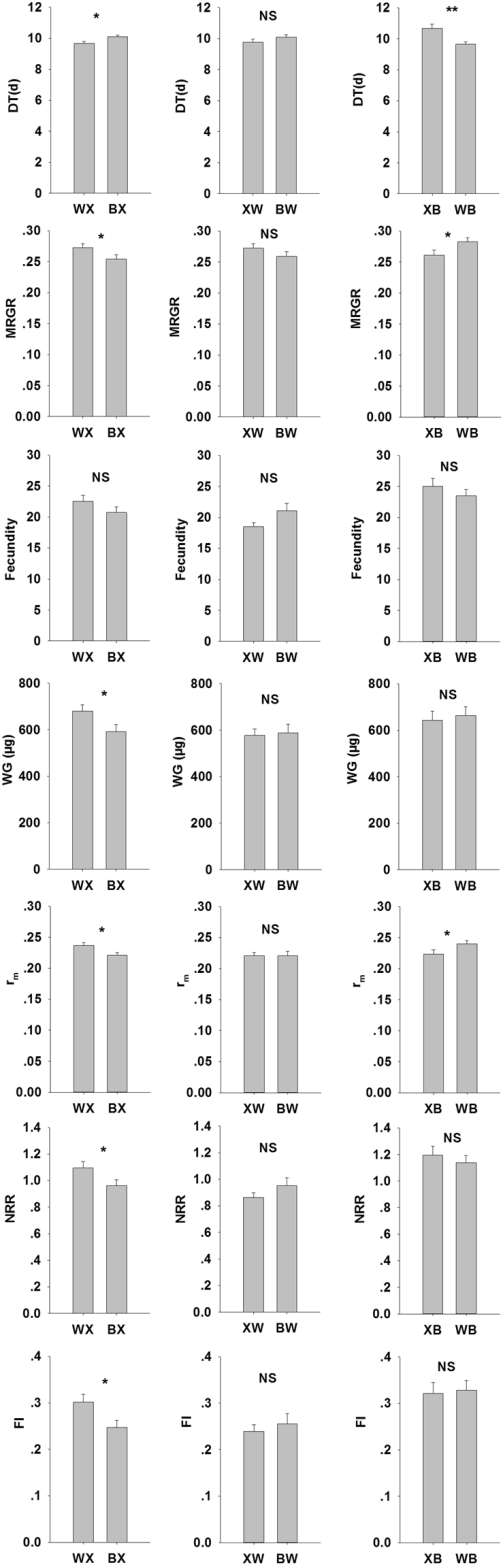
The life-history trait values of different population offspring aphids on same transition variety.

The comparisons of aphid offspring life-history traits for different populations on the same transition variety, WX vs. BX, XW vs. BW and XB vs. WB are shown in Fig. 4.

The WW2730 population had a significantly shorter DT, and greater MRGR, WG, r_m_, NRR and FI as compared with those of the Batis population on Xiaoyan22. The life-history trait on WW2730 were not significantly different between the Xiaoyan22 and Batis populations. On Batis, the Xiaoyan22 population had significantly longer DT and lower MRGR and r_m_ than those of the WW2730 population.

**Figure 5:**
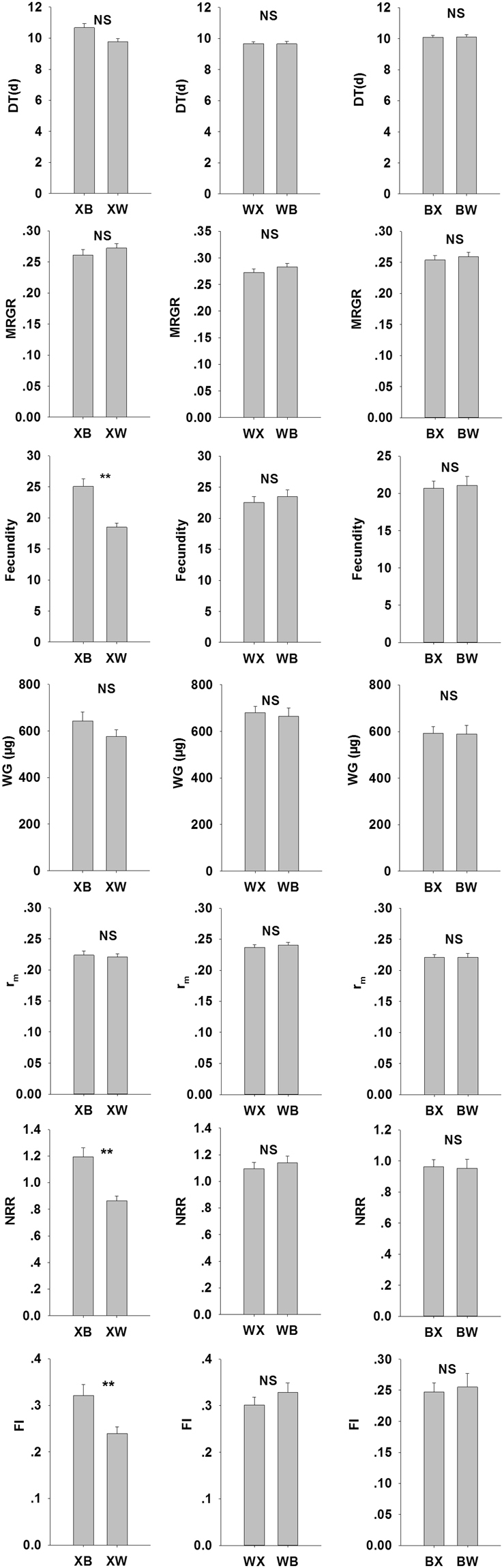
The life-history trait values of same population offspring aphids on different transition variety.

The comparisons of life-history trait values for same population on different transition varieties XB vs. XW, WX vs. WB and BX vs. BW are depicted in Fig. 5.

The Xiaoyan22 population showed greater fecundity, NRR, and FI on Batis than those on WW2730. The life-history traits of the WW2730 population were not significantly different between Xiaoyan22 and Batis, and those of the Batis population were not significantly different between Xiaoyan22 and WW2730.

**Figure 6:**
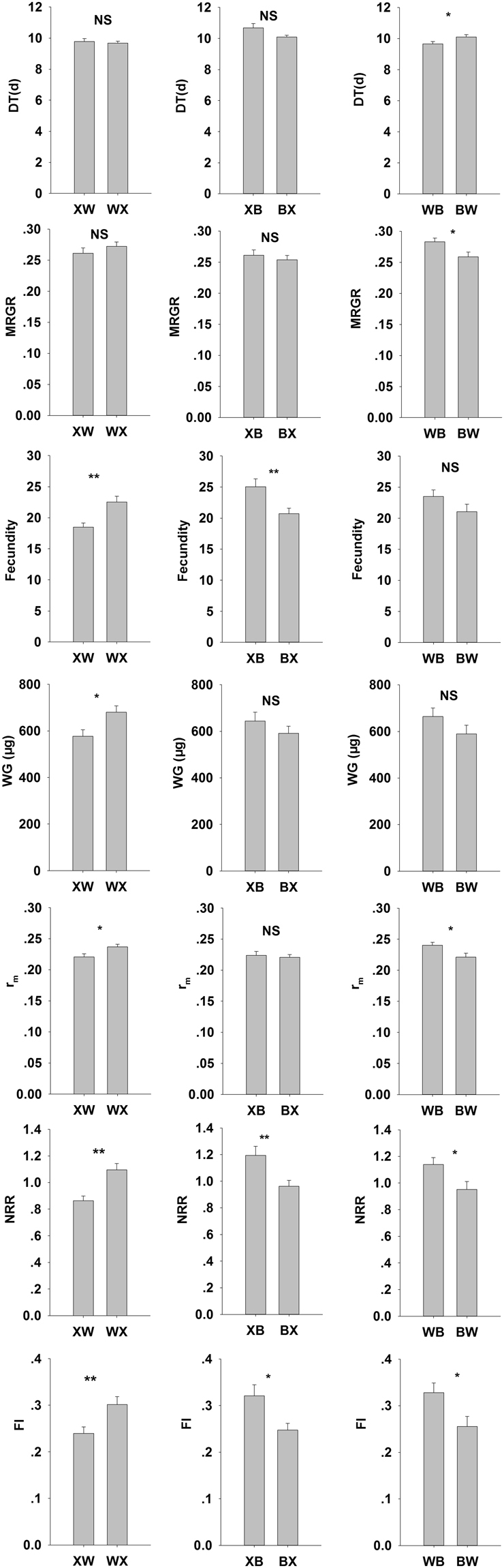
The life-history trait values of different populations offspring aphids reciprocally shifted host.

The results of different populations reciprocally shifted host varieties are shown in Fig. 6.

The fecundity, WG, r_m_, NRR and FI of the WW2730 population were significantly greater on transition variety Xiaoyan22 than that of the Xiaoyan22 population on the transition variety WW2730. The fecundity, NRR and FI of the Xiaoyan22 population on the transition variety Batis were significantly greater than that of the Batis population on the transition variety Xiaoyan22. The MRGR, r_m_, NRR and FI of the WW2730 population on the transition variety Batis were greater than that of the Batis population on the transition variety WW2730.

## Discussion

### Maternal effects of conditioning wheat varieties (maternal diet) on offspring traits

Besides individual genes, the ambient environmental conditions of the maternal generation might affect the phenotype of the offspring. These maternal effects have been found in different clones of the aphids *Myzus persicae*, *A. pisum*, *R. padi* and *A. nerii*^3,10,15,16,32–35^.

In *S. avenae*, the feeding experience of the mother aphids often influences the subsequent performance of their offspring. For instance, the time from birth until the onset of reproduction and the r_m_ were found to be greatly influenced by clonal factors in three *S. avenae* clones originating from three regions in French Oceania^21^. The mean weight and fecundity were found to be greatly influenced by clonal factors in clones originally collected from eight wheat and eight cocksfoot stands around Hampshire, UK^36^. The development and pre-reproductive duration and fecundity differed between a population from Spain and one from England^33^. In another study, the r_m_ was greatly influenced by clonal factors in four apterous clones from different parts of England^37,38^. In the present study, we assessed the DT, WG, and r_m_ in response to transition varieties in three acclimatized populations of *S. avenae*. The Xiaoyan22 population had longer DTs and lower r_m_ values than those of the WW2730 population, and both populations had less WG than that of the Batis population. There were significant interaction effects of the conditioning and transition wheat varieties on the DT, MRGR, and fecundity. This implies that these three parameters of *S. avenae* in response to the transition varieties differed depending on the conditioning variety. The presence of maternal effects on the aphids offspring is required to evaluate the interaction term of the model and make specific comparisons of the means originating from this interaction. The comparisons between the same acclimatized population on different transition varieties would indicate the extent of the actual maternal effects. The results of the three comparisons, XB vs. XW, WX vs. WB and BX vs. BW, showed that the WW2730 population had better fitness than that of the Batis population on Xiaoyan22; it also had better fitness than that of the Xiaoyan22 population on Batis.

### Influence of transition varieties on aphid offspring traits

Maternal effects influence the individual growth and development of *A. nerii*, and might affect their population dynamics; however, the current environment more strongly affects the population dynamics^15^. In two generalist and two specialist neotropical beetles *Cephaloleia* spp. (Coleoptera: Chrysomelidae), the survival of the larvae was worse, while that of the adults was better, or at least similar on the novel host compared to that on the native host^39–41^. The response of *R. padi* to maternal effects is indirect, only evidenced by wing-type, while other biological parameters are more affected by the current dietary host of the offspring^10^. In the Chinook salmon *Oncorhynchus tshawytscha*, response to maternal effects is evidenced by the egg size; however, these maternal effects decrease with an increase in temperature^42^. The spruce budworm *Choristoneura fumiferana* (Clem.) responds to chronic nutritional stress via phenotypic plasticity of food utilization traits rather than through maternal effects when its host plants changes^5^. Our results are similar to these reports where the conditioning varieties only directly influenced on WG, and influenced the DT, MRGR, and fecundity through the interaction effect with the transition varieties. The transition environment affected the traits related to fecundity and population parameters (e.g., fecundity, r_m_, NRR, and FI) of *S. avenae* feeding on transition varieties. Comparing the life-history traits of the same acclimatized population on different transition wheat varieties can provide clues on whether the resistance and susceptible patterns hold independently of the conditioning wheat variety. We expected XB vs. XW and WX vs. WB to be significantly different, but this was found only in first the former (XB vs. XW). Furthermore, we also did not find a significantly difference for the BX vs. BW comparison. Thus, it appears that maintaining a stock aphid population on a susceptible variety and then transferring it to novel varieties is not a better way to finely distinguish resistance levels as we always used to evaluate the resistance of wheat varieties to aphids. However, from the comparison results of reciprocal transfer (Fig. 6), we could conclude that WW2730 is a more resistant variety than Xiaoyan22, and Batis is a relatively susceptible variety.

### Influence of host resistance on *S. avenae*

Transfer to a novel host plant is the primary strategy when a herbivore faces the threat of food shortage. Many insects show asymmetrical differences or unfitting responses, and might not even survive after transfer to novel hosts belonging to different families, genera, species, or closely related taxa, such as several aphid species, *M. persicae*, *Schizaphis graminum*, *Macrosiphum avenae*, *R. maidis*, *Aphis gossypii*, and the whitefly *Bemisia tabaci*, and *Trialeurodes vaporariorum*^43–48^. Different clones of *S. aven*ae collected from wheat and cocksfoot stands were reported to perform less well on the alternate host than they did on the original host^36^. However, in the present study, we found that both the conditioning and transition wheat varieties asymmetrically affected the biological parameters of *S. avenae* offspring after they shifted between host wheat varieties with different resistance traits. We found that when *S. avenae* individuals were transferred from the susceptible Batis to the resistant WW2730 or Xiaoyan22, the MRGR decreased. When *S. avenae* individuals were transferred from the resistant Xiaoyan22 to the susceptible Batis, the DT and fecundity increased. On Batis, the DT of the Xiaoyan22 population was longer than that on both Batis and WW2730 populations; MRGRs were also lower than those on the Batis population. Our results showed interactive effects on DT, fecundity, and MRGR in *S. avenae*. The most likely explanation is that host wheat resistance induced an interaction between the conditioning and transition wheat varieties. Our results are consistent with previous findings on strong population-morph interactions in *S. avenae* and *R. padi*^10,21^. When *S. avenae* individuals were transferred from a resistant variety to a susceptible line of einkorn wheat (*Triticum monococcum*), embryo growth and the number of matured embryos increased within the first 10 days of adult life, which compensated for their poor nymphal growth on the resistant variety. Conversely, when *S. avenae* individuals were transferred from susceptible to resistant plants, most aphids died, although most advanced embryos matured and were born, and subsequent embryo growth was quickly reduced within the first week^22^.

### Individual-level and population-level hierarchical traits

We found that DT and WG, which determined the duration from nymph birth to adult emergence, were significantly different among conditioning varieties. Meanwhile, the fecundity, NRR and FI, which are based on the entire lifespan, were significantly different among transition varieties. Hence, the conditioning wheat varieties affected the life-history traits of *S. avenae* related to individual growth and development, while transition wheat varieties affected the traits related to fecundity and population parameters of the aphids.

The equations for MRGR have denominators of DT, Wn and Wa. The longest DT was on the Xiaoyan22 population; however, the highest WG was on the Batis population. This suggest that MRGR is not significant among conditioning or transition varieties. The r_m_ is widely used to determine the antibiosis effects of plants to pests^49,50^. Our results show that the r_m_ was significantly different in both conditioning and transition varieties. As for the NRR and FI, the r_m_ can be ranked as WW2730 < Xiaoyan22 < Batis on the transition varieties, and these parameters were negative correlated with the resistance level. However, the NRR and FI were significantly different between Xiaoyan22 and Batis, but r_m_ was not. This suggest that NRR and FI possible might serve as alternate parameters to measure resistance.

In conclusion, when the maternal aphids feeding on conditioning wheat varieties for over 3 months, obvious maternal effects had an effects on the offspring of *S. avenae*. The conditioning and transition maternal host wheat varieties affected the life-history traits of *S. avenae* related to individual growth and development, and transition wheat varieties affected the traits related to fecundity and population parameters of the aphids. The resistance of wheat varieties could induce an interaction between the conditioning and transition wheat varieties to influence the growth, development, reproduction, and even population dynamics of aphids.

The field spatial distribution pattern of the English grain aphids is the aggregated distribution in early spring with immigration of the wing aphids, and became progressively homogeneously distributed with the aphid dispersion in the later^51^. That means compared with large-scale planting of a single wheat variety, inlaid or strip planting different wheat varieties with different resistance traits to aphids in the field maybe have a greater effect on aphid at individual, even population level. However, further studies are required to determine whether this technique could inhibit pest population growth and diffusion in the field.

## References

[1] Scheiner SM (1993). Genetics and evolution of phenotypic plasticity. Annu Rev Ecol Syst..

[2] Gotthard K, Nylin S (1995). Adaptive plasticity and plasticity as an adaptation: a selective review of plasticity in animal morphology and life history. Oikos..

[3] Via S (1995). Adaptive phenotypic plasticity: consensus and controversy. Trends Ecol Evol..

[4] Agrawal AA (2001). Phenotypic plasticity in the interactions and evolution of species. Science..

[5] Quezada-García R, Fuentealba Á, Bauce É (2017). Phenotypic variation in food utilization in an outbreak insect herbivore. Insect Sci..

[6] Mousseau TA, Dingle H (1991). Maternal effects in insect life histories. Ann. Rev. Entomol..

[7] Wolf JB, Brodie ED (1998). The coadaptation of parental and offspring characters. Evolution..

[8] Ezard THG, Prizak R, Hoyle RB (2014). The fitness costs of adaptation via phenotypic plasticity and maternal effects. Funct Ecol..

[9] Mousseau TA, Fox CM (1998). The adaptive significance of maternal effects. Trends Ecol Evol..

[10] Hu X-S (2016). Effects of maternal diet on offspring fitness in the bird cherry-oat aphid. Ecol Entomol..

[11] Dixon, A. F. G. Aphid Ecology: An Optimization Approach, 2nd edn. Chapman & Hall, London, U.K. (1998).

[12] Hales DF (2002). Lack of detectable genetic recombination on the X chromosome during the parthenogenetic production of female & male aphids. Genet Res..

[13] Hales DF, Tomiuk J, Wöhrmann K, Sunnucks P (1997). Evolutionary and genetic aspects of aphid biology: a review. Euro J Entomol..

[14] Caballero PP, Ramirez CC, Niemeyer HM (2001). Specialisation pattern of the aphid *Rhopalosiphum maidis* is not modified by experience on a novel host. Entomol Exp Appl..

[15] Zehnder CB, Hunter MD (2007). A comparison of maternal effects and current environment on vital rates of *Aphis nerii*, the milkweed-oleander aphid. Ecol Entomol..

[16] McLean AH, Ferrari J, Godfray HCJ (2009). Effects of the maternal and pre-adult host plant on adult performance and preference. Ecol. Entomol..

[17] Leather SR (1982). Preliminary studies on the effect of host age and aphid generation on the reproduction and survival of the bird cherry-oat aphid, *Rhopalostphum padi* (L.). Ann Agr Fenn..

[18] Dixon, A. F. G. Parthenogenetic reproduction and the rate of increase in aphids. In: Minks AK and Harrewijn P. Aphids, their biology, natural enemies and control (Vol. 2A)(Eds). Elsevier: Amsterdam, Oxford, New-York, Tokyo P: 269–287(1987).

[19] Kieckhefer RW, Gellne JL (1992). Yield losses in winter wheat caused by low-density cereal aphid populations. Agr J..

[20] Liu X-F (2014). Tripartite interactions of barley yellow dwarf virus, Sitobion avenae and wheat varieties. PLoS One..

[21] Simon JC, Dedryver CA, Pierre JS, Tanguy S, Wegorek P (1991). The influence of population and morph on the parameters of intrinsic rate of increase in the cereal aphids *Sitobion avenae* and *Rhopalosiphum padi*. Entomol Exp App..

[22] Cailaud CM, Dedryyver CA, Simon JC (1994). Development and reproductive potential of the cereal aphid *Sitobion avence* on resistant wheat lines (*Triticum monococcum*). Ann Appl Bio..

[23] Xu Z-H (2011). Discovery of English grain aphid (Hemiptera: Aphididae) biotypes in China. J Econ Entomol..

[24] Gao S-X, Liu D-G, Chen H, Meng X-X (2014). Fitness traits and underlying genetic variation related to host plant specialization in the aphid *Sitobion avenae*. Insect Sci..

[25] Xin J-J, Shang Q-L, Desneux N, Gao X-W (2014). Genetic diversity of *Sitobion avenae* (Homoptera: Aphididae) populations from different geographic regions in China. PLoS ONE..

[26] Liu Y, Ni H-X, Sun J-R, Hu C (2001). The effects of wheat varieties resistant to aphids on the population of *Sitobion avenae* (Fabricius) and parasitization as well as development of its parasitic wasp *Aphidius avenae* Haliday. Acta Phytophy Sin..

[27] Hesler LS, Tharp CI (2005). Antibiosis and antixenosis to Rhopalosiphum padi among triticale accessions. Euphytica..

[28] Hu X-S (2013). The resistance and correlation analysis to three species of cereal aphids (Hemiptera: Aphididae) on 10 wheat varieties/lines. J Econ Entomol..

[29] Hu XS, Zhao HY, Hu ZQ, Li DH, Zhang YH (2008). EPG comparison of *Sitobion avenae* (Fab.) feeding behavior on three wheat varieties. Agr Sci China..

[30] Thieme T, Heimbach U (1996). Development and reproductive of cereal aphids (Homoptera: Aphididae) on winter wheat cultivals. WPRS Bull..

[31] Gorur G, Lomonaco C, Mackenzie A (2005). Phenotypic plasticity in host-plant specialisation in *Aphis fabae*. Ecol Entomol..

[32] Eggers-Schumacher HA (1983). A comparison of the reproductive performance of insecticide resistant and susceptible clones of *Myzus persicae*. Entomol Exp Appl..

[33] Pons X, Tatchell GM (1995). Drought stress and cereal aphid performance. Ann Appl Bio..

[34] Blackman, R. L. & Eastop, V. F. Aphids on theWorld’s Crops: An Identification and Information Guide. Chichester: John Wiley & Sons Ltd. 466 p (2000).

[35] Glinwood R, Pettersson J (2000). Host choice and host leaving in *Rhopalosiphum padi* (Hemiptera: Aphididae) emigrants and repellency of aphid colonies on the winter host. Bull Entomol Res..

[36] De Barro PJ, Sherratt TN, David O, Maclean N (1995). An investigation of the differential performance of clones of the aphid *Sitobion avenae* on two host species. Oecologia..

[37] Khan M, Port G (2008). Performance of clones and morphs of two cereal aphids on wheat plants with high and low nitrogen content. Entomol Sci..

[38] Khan M (2005). Use of RAPD-PCR to determine the genetic variation of four different clones of English grain aphid, *Sitobion avenae* (F.) (Homoptera: Aphididae). Bangladesh. J Entomol..

[39] García-Robledo C, Horvitz CC (2011). Experimental demography and the vital rates of generalist and specialist insect herbivores on native and novel host plants. J Anim Ecol..

[40] García-Robledo C, Horvitz CC (2012). Jack of all trades masters novel host plants: positive genetic correlations in specialist and generalist insect herbivores expanding their diets to novel hosts. J Evol Bio..

[41] García-Robledo C (2017). Experimental assemblage of novel plant–herbivore interactions: ecological host shifts after 40 million years of isolation. Biotropica..

[42] Thorn MW, Morbey YE (2018). Egg size and the adaptive capacity of early life history traits in Chinook salmon (*Oncorhynchus tshawytscha*). Evol Appl..

[43] Apablaza JU, Robinson AG (1967). Effects on three species of grain aphids (Homoptera: aphididae) reared on wheat, oats or barley and transferred as adults to wheat, oats or barley. Entoml Exp Appl..

[44] Zhao H-Y, Wang S-Z, Yuan F, Dong Y-C (1997). Study on the effects of host altercation on some ecological characteristics of green peach aphid under different temperature conditions. Acta Phytophy Sin..

[45] Zhang X-X, Zhao J-Y, Zhang G-X, Chen X-F (2001). Studies on population adaptation and differentiation of *Aphis gossypii* Glover among host plant transplantation. Acta Ecol Sin..

[46] Liu X-D, Zhai B-P, Zhang X-X, Fang J (2004). The fitness of the host biotype of cotton aphid, *Aphis gossypii*, to summer host plants. Acta Ecol Sin..

[47] Liu X-D, Zhai B-P, Zhang X-X, Lu Y (2004). Differentiation in morphometrics and ecological adaptability of cotton and cucumber biotypes of the cotton aphid *Aphis gossypii*(Homoptera: Aphididae). Acta Ecol Sin..

[48] Lei F, Zhang G-F, Wan F-H, Ma J (2006). Effects of plant species switching on dynamics of trehalose and trehalase activity of *Bemisia tabaci* B-biotype and *Trialeurodes vaporariorum*. Acta Entom Sin..

[49] Wojciechowicz–Zytko E, van Emden HF (1995). Are mean relative growth rate and intrinsic rate of increase likely to show a correlation in plant resistance studies?. J Appl Entomol..

[50] Hu X-S (2015). Testing the fecundity advantage hypothesis with *Sitobion avenae, Rhopalosiphum padi*, and *Schizaphis graminum* (Hemiptera: Aphididae) feeding on ten wheat accessions. Sci Rep..

[51] Fievet V, Dedryver C-A, Plantegenest M, Simon J-C, Outreman Y (2007). Aphid colony turn-over influences the spatial distribution of the grain aphid *Sitobion avenae* over the wheat growing season. Agr Forest Entomol..

